# The association of platelet to white blood cell ratio with diabetes: a nationwide survey in China

**DOI:** 10.3389/fendo.2024.1418583

**Published:** 2024-06-18

**Authors:** Fanglin Liu, Tianhong Wang, Siman Wang, Xiumei Zhao, Yusi Hua

**Affiliations:** ^1^ Department of Anesthesiology, West China Hospital, Sichuan University/West China School of Nursing, Sichuan University, Chengdu, China; ^2^ Department of Anesthesiology, West China Hospital, Sichuan University, Chengdu, China; ^3^ Operating Room, West China Hospital, Sichuan University/West China School of Nursing, Sichuan University, Chengdu, China

**Keywords:** platelet, white blood cell, diabetes, prediabetes, CHARLS

## Abstract

**Background:**

Inflammation is integral to diabetes pathogenesis. The novel hematological inflammatory biomarker, platelet to white blood cell ratio (PWR), is linked with various conditions such as chronic kidney disease and stroke. However, the association of this novel clinical indicator with diabetes still remains unclear, which is investigated in this study.

**Materials and Methods:**

A total of 10,973 Chinese participants were included and grouped according to the tertiles of PWR (T1, T2, and T3 groups). Diagnosis of prediabetes and diabetes adhered to American Diabetes Association criteria. Binary logistic regression was adopted to assess the relationship between PWR and both diabetes and prediabetes. The dose-response relationship of PWR and diabetes was examined using restricted cubic spline regression. Subgroup and interaction analyses were conducted to investigate potential covariate interactions.

**Results:**

Individuals with higher PWR had better lifestyles and lipid profiles (all *P* < 0.05). After adjusting for all the covariates, the T2 group had a 0.83-fold (95% CI: 0.73–0.93, *P* < 0.01) risk of diabetes and that for the T3 group was 0.68-fold (95% CI: 0.60–0.78. *P* < 0.001). Dose-response analysis identified non-linear PWR-diabetes associations in the general population and females (both *P* < 0.05), but absent in males. Participants with prediabetes in the T2 and T3 groups had lower risks of diabetes (OR = 0.80 for the T2 group, *P* < 0.001 and 0.68 for the T3 group, *P* < 0.001) in the full models. All the sensitivity analysis support consistent conclusions.

**Conclusions:**

An increase in PWR significantly correlates with reduced diabetes risks. A non-linear PWR-diabetes relationship exists in the general population and females, but not in males. The correlation between PWR and diabetes indicates that PWR holds potentials in early identification and prevention of diabetes.

## 1 Introduction

Diabetes, a chronic condition, is ranked as the ninth leading cause of death (1, 2), making it one of the most important health challenges in 21st century (3). It poses a long-term threat to human health and quality of life, causing millions of deaths globally each year. The economic consequences of diabetes and its associated complications continue to grow, with approximately 12% of the global healthcare expenditure allocated to managing this condition, amounting to a staggering $727 billion (4). Current projections indicate that diabetes affects 9% of the worldwide populace, while an additional 7.3% show signs of impaired glucose tolerance (4). Among those with impaired glucose tolerance (prediabetes), an alarming 5% to 10% are anticipated to progress to diabetes each year (5). Prediabetes represents the second stage in the progression of diabetes, following the stage of being at high risk and preceding the development of full-fledged diabetes (6). It can be defined based on impaired fasting glucose or impaired glucose tolerance levels, recognizing individuals with an elevated risk of developing type 2 diabetes (7). Observational studies have revealed connections between prediabetes and the initial stages of small fiber neuropathy, nephropathy, diabetic retinopathy, as well as a heightened susceptibility to macrovascular disease (7). Diabetic patients face a 2- to 6-fold heightened susceptibility to cardiovascular diseases in contrast to individuals without diabetes, making them more vulnerable to conditions such as cardiac insufficiency, peripheral vascular disease, and coronary artery disease, leading to a significant increase in cardiovascular mortality (8). Diabetes is also linked to higher risks of liver ailments, such as nonalcoholic fatty liver disease, chronic liver disease and hepatocellular carcinoma, contributing to its prominence as the seventh most common cause of mortality in the United States in 2017 (9). Initiating screening protocols for prediabetes and type 2 diabetes among asymptomatic adults can facilitate timely identification, diagnosis, and intervention, thereby enhancing health outcomes.

Insufficient pancreatic β-cell function and insulin resistance are crucial factors contributing to the onset and progression of diabetes (10, 11). One recent research has revealed a close association between chronic low-grade inflammation and the onset of obesity, metabolic syndrome, and diabetes (12). Chronic inflammation can lead to β-cell apoptosis (13), and induce metabolic reprogramming in the liver, adipose tissue, skeletal muscle, and other tissues (14), resulting in insulin resistance and peripheral hyperinsulinemia. The neutrophil-to-lymphocyte ratio (NLR), platelet-to-lymphocyte ratio (PLR), and monocyte-to-lymphocyte ratio (MLR) are indicators reflecting systemic inflammation levels, which have been demonstrated to be closely associated with the progression and prognosis of various cancers (15, 16), cardiovascular diseases (17, 18), and autoimmune diseases (19, 20). Additionally, studies have found correlations between the NLR, PLR, and MLR and the onset of diabetes and its complications (21–23). The platelet to white blood cell ratio (PWR) is a novel hematological marker of inflammation that has recently gained increased attention (24). The PWR has emerged as a significant predictor of clinical outcomes across a spectrum of diseases, including chronic kidney disease (25), cirrhosis (24), acute promyelocytic leukemia (26), ischemic stroke (27), intracerebral hemorrhage (28), and pancreatic cancer (29). However, the correlation between PWR and diabetes has not been established.

Therefore, in this study, utilizing the public data from the China Health and Retirement Longitudinal Study (CHARLS), we aimed to investigate the association between PWR and diabetes, with the goal of providing novel observational indicators for early screening and prevention of diabetes.

## 2 Materials and methods

### 2.1 Study population and designs

CHARLS, a nation-wide and dynamic project, aimed to survey the social, economic, and health status, as well as long-term changes in healthcare utilization and insurance coverage among Chinese residents aged 45 and older. This project was established in 2011 and participants were enrolled and followed up in 2013, 2015, 2018 and 2020. As a large-scale national survey, CHARLS employed a multistage stratified probabilities proportional sampling design to obtain a representative sample covering 150 counties and 450 villages. A detailed description of the study design and data processing methods can be found in prior publications (30). CHARLS received approval from Peking University’s ethical review board (IRB number:00001052–11014). Prior to each interview, written or oral consent was secured from respondents.

**Figure 1:**
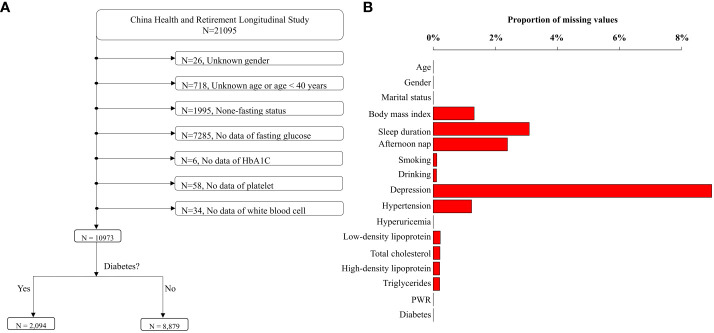
Flowchart of data cleansing and proportions of missing values. **(A)** depicts the process of data cleansing. After data cleansing, 10,973 individuals were remained. The proportions of missing values in the cleaned dataset were displayed in **(B)**.

In our study, CHARLS 2015 was the latest datasets used to explore the relationship between PWR and diabetes, as blood and urine biomarkers were available in 2011 and 2015 cycles. Therefore, the cross-sectional design using CHARLS 2015 can provide sufficient information on the epidemiological status of chronic diseases and underlying biological mechanisms. We used CHARLS 2015 for examining our hypothesis. Briefly, there were 21,095 participants included in the CHARLS 2015. Participants were excluded due to unknown sex, unknown age or age < 40 years, none-fasting status, and missing data on fasting glucose, HbA1C, platelet, and white blood cell. Ultimately, the analysis incorporated 10,973 participants, comprising 2,094 diabetics cases and 8,879 healthy individuals (**Figure 1A**). Since the proportion of missing data for other covariates ranged 0% to 8.98% (**Figure 1B**), which was deemed acceptable, we imputed the missing variables using multivariate imputation based on random forest (31). We utilized the imputed datasets as the main research datasets, while the original dataset was regarded as the validated datasets.

### 2.2 Assessment of PWR, prediabetes, and diabetes

PWR was derived from platelets (10^9^/L)/white blood cells (10^9^/L) (25). The diagnosis of diabetes was established upon the elevation of one or more diagnostic markers, including fasting glucose (≥ 126 mg/dL), HbA1C (≥ 6.5%), self-reported medical history, or the administration of anti-diabetic drugs (32). In non-diabetic subjects, prediabetes was delineated by fasting glucose levels of 100–126 mg/dL or HbA1C values of 5.7%-6.5%, while normoglycemia was defined by fasting glucose below 100 mg/dL and HbA1C less than 5.7%.

### 2.3 Collection of blood biomarkers

Venous blood samples were procured from each participant in the morning following an overnight fast. This procedure, adhering to standard protocols, was executed by professional nurses and involved the collection of three blood tubes per participant (33). The complete blood count analysis was conducted using the first 2 mL tube on an automated analyzer. The second tube, containing 6 mL of whole blood, was utilized for the quantification of blood lipids, glucose, and et al. The third tube was employed for determining HbA1C. A detailed protocol for processing and assessing blood biomarkers can be found in an established study (33).

### 2.4 Definition of covariates

Certain covariates were included in our analysis, comprising demographics, lifestyles, health examinations, and the histories of chronic diseases. Demographic variables encompassed age, educational status (categorized as literate or illiterate), marital status (classified as married/cohabitating or other) and gender (male or female). Illiterate respondents were defined as those lacking elementary school education. Lifestyle determinants encompassed sleep duration (categorized as < 6h, 6–8h, and >8h), nap (yes or no), smoking habits (current, never, or former), and frequency of alcohol consumption (classified as more than once per month, less than once per month, or never). Chronic diseases included hypertension, depression and hyperuricemia. BMI, hypertension and depression were assessed and grouped as one previous study did (31). Hyperuricemia and blood lipid panel, including low-density lipoprotein (LDL, mg/dL), total cholesterol (TC, mg/dL), triglyceride (TG, mg/dL), and high-density lipoprotein (HDL, mg/dL), were included based on the previous study (32).

### 2.5 Statistical analysis

To quantify the difference in PWR levels, PWR was divided into tertiles (T1, T2, and T3). Comparative analysis across tertiles was conducted utilizing ANOVA, Kruskal-Wallis test, or Chi-square test, contingent on the variable classifications. The correlation of PWR with diabetes was assessed using logistic regression. Four nested models were completed in order. Model 1 served as the unadjusted model. Model 2 incorporated adjustments for age, gender, marital status, educational attainment, and BMI. Model 3 extended these adjustments to include lifestyle factors such as smoking and drinking habits, sleep, and nap. Model 4 was further adjusted for chronic diseases, such as depression, hypertension, and hyperuricemia, as well as lipids panel, such as LDL, HDL, TC, and TG. A trend test was executed to evaluate the linear relationship between PWR and diabetes.

To corroborate the stability of the observed association, several sensitivity analyses were conducted. First, the continuous form of PWR was included in the full adjusted model instead of the tertiles to verify the linear association between PWR and diabetes. We also modeled the regression model based on the median and quantiles of PWR. Second, a restricted cubic spline (RCS) with three knots was employed to delineate the nonlinear relationship of PWR with diabetes risk, and the test of nonlinearity was performed using the Wald ratio test. Third, we conducted subgroup analysis to identify potential vulnerable populations and examine joint effects. The interactive effects were explored by constructing a multiplication interaction term. Fourth, the data without interpolation was used to compare the findings from interpolated data.

Data analysis was conducted utilizing R software (version 4.0.2). A two-sided *P* value of < 0.05 was considered statistically significant.

## 3 Results

### 3.1 Characteristics of sample

The final analysis incorporated a total of 10,973 participants (**Figure 1A**). The characteristics of participants across PWR tertiles were shown in **Table 1** (missing values not interpolated). The  PWR  ranges for Tertile 1, Tertile 2, and Tertile 3 were 1.50 to 29.50, 29.51 to 40.20, and 40.21 to 280.68, respectively. Our results revealed significant differences across tertiles in various demographic and health-related variables. Participants in higher PWR tertiles were generally younger, with the average age decreasing from Tertile 1 (61.08 years) to Tertile 3 (58.81 years) (*P* < 0.001). Gender distribution also varied significantly, with a higher proportion of females in Tertile 3 (68.23%) compared to Tertile 1 (42.03%) (*P* < 0.001).

**Table 1:** Characteristics of included participants.

Covariates	Tertiles of PWR			Total N = 10973	*P*
	T1 N = 3659	T2 N = 3659	T3 N = 3659		
**Age (years)**	61.08 ± 10.12	60.29 ± 9.95	58.81 ± 9.79	60.06 ± 10.00	<0.001
Gender					<0.001
Male	2121 (57.97%)	1766 (48.29%)	1162 (31.77%)	5049 (46.01%)	
Female	1538 (42.03%)	1891 (51.71%)	2495 (68.23%)	5924 (53.99%)	
Marital status					0.412
Married/cohabitating	3059 (83.60%)	3041 (83.16%)	3015 (82.44%)	9115 (83.07%)	
Others	600 (16.40%)	616 (16.84%)	642 (17.56%)	1858 (16.93%)	
BMI (Kg/m^2^)					0.025
<18.5	207 (5.75%)	199 (5.52%)	191 (5.27%)	597 (5.51%)	
18.5–24.0	1688 (46.88%)	1633 (45.29%)	1774 (48.99%)	5095 (47.05%)	
24.0–28.0	1186 (32.94%)	1283 (35.58%)	1191 (32.89%)	3660 (33.80%)	
≥28.0	520 (14.44%)	491 (13.62%)	465 (12.84%)	1476 (13.63%)	
Cigarette consumption					<0.001
Current smoker	1245 (34.06%)	1038 (28.42%)	694 (19.00%)	2977 (27.16%)	
Non-smoker	1828 (50.01%)	2126 (58.20%)	2615 (71.60%)	6569 (59.94%)	
Ex-smoker	582 (15.92%)	489 (13.39%)	343 (9.39%)	1414 (12.90%)	
Alcohol consumption					<0.001
Drink more than once a month	1080 (29.56%)	1011 (27.66%)	823 (22.53%)	2914 (26.59%)	
Drink less than once a month	314 (8.60%)	353 (9.66%)	304 (8.32%)	971 (8.86%)	
None of These	2259 (61.84%)	2291 (62.68%)	2526 (69.15%)	7076 (64.56%)	
Sleep duration (hours)					0.904
0–6	1802 (50.60%)	1801 (50.90%)	1766 (49.97%)	5369 (50.49%)	
6–8	1426 (40.04%)	1395 (39.43%)	1421 (40.21%)	4242 (39.89%)	
>8	333 (9.35%)	342 (9.67%)	347 (9.82%)	1022 (9.61%)	
Afternoon nap					0.012
No	1428 (39.97%)	1447 (40.53%)	1541 (43.20%)	4416 (41.23%)	
Yes	2145 (60.03%)	2123 (59.47%)	2026 (56.80%)	6294 (58.77%)	
Depression					<0.001
No	2209 (66.64%)	2308 (69.06%)	2146 (64.43%)	6663 (66.71%)	
Yes	1106 (33.36%)	1034 (30.94%)	1185 (35.57%)	3325 (33.29%)	
Hypertension					<0.001
No	2061 (57.04%)	2097 (58.10%)	2334 (64.56%)	6492 (59.91%)	
Yes	1552 (42.96%)	1512 (41.90%)	1281 (35.44%)	4345 (40.09%)	
Hyperuricemia					<0.001
No	3141 (85.84%)	3255 (89.01%)	3377 (92.34%)	9773 (89.06%)	
Yes	518 (14.16%)	402 (10.99%)	280 (7.66%)	1200 (10.94%)	
LDL (mg/dL)					<0.001
≤120	2836 (77.78%)	2720 (74.44%)	2679 (73.44%)	8235 (75.22%)	
>120	810 (22.22%)	934 (25.56%)	969 (26.56%)	2713 (24.78%)	
Total cholesterol (mg/dL)					<0.001
≤200	2669 (73.18%)	2554 (69.90%)	2491 (68.28%)	7714 (70.45%)	
>200	978 (26.82%)	1100 (30.10%)	1157 (31.72%)	3235 (29.55%)	
Reduced HDL					<0.001
No	2363 (64.79%)	2280 (62.38%)	2156 (59.10%)	6799 (62.09%)	
Yes	1284 (35.21%)	1375 (37.62%)	1492 (40.90%)	4151 (37.91%)	
Elevated triglycerides					0.001
No	2332 (63.94%)	2382 (65.17%)	2477 (67.90%)	7191 (65.67%)	
Yes	1315 (36.06%)	1273 (34.83%)	1171 (32.10%)	3759 (34.33%)	
**Platelets (×10^9^/L)**	152.74 ± 54.71	206.88 ± 47.88	256.63 ± 82.54	205.41 ± 76.37	<0.001
**White blood cell (×10^9^/L)**	6.84 ± 2.83	6.00 ± 1.36	5.04 ± 1.27	5.96 ± 2.09	<0.001
**PWR index**	22.59 ± 5.39	34.58 ± 3.01	51.70 ± 13.47	36.29 ± 14.69	<0.001
**PWR index range**	1.50–29.50	29.51–40.20	40.21–280.68	1.50–280.68	–
**Fasting glucose (mg/dL)**	102.68 ± 33.28	101.07 ± 31.04	97.75 ± 25.88	100.50 ± 30.30	<0.001
**Glycated hemoglobin (%)**	6.04 ± 1.12	5.98 ± 0.98	5.92 ± 0.85	5.98 ± 0.99	<0.001
Diabetes					<0.001
No	2840 (77.62%)	2952 (80.72%)	3087 (84.41%)	8879 (80.92%)	
Yes	819 (22.38%)	705 (19.28%)	570 (15.59%)	2094 (19.08%)	

PWR is defined as the amount of platelet (10^9^/L) divided by white blood cells (10^9^/L). The participants were grouped according to the tertiles of PWR. The others group in marital status refers to the divorced/separated/widowed. BMI, body mass index; HDL, high-density lipoprotein; LDL, low-density lipoprotein; PWR, platelet to white blood cell ratio; T, tertile.

Health behavior differences were notable, with higher PWR tertiles showing lower proportions of current smoking and alcohol consumption. For instance, the proportion of current smokers decreased from Tertile 1 (34.06%) to Tertile 3 (19.00%) (*P* < 0.001), and those who drank alcohol more than once a month decreased from Tertile 1 (29.56%) to Tertile 3 (22.53%) (*P* < 0.001).

Metabolic health indicators such as fasting glucose and glycated hemoglobin levels were also more favorable in higher PWR tertiles. Fasting glucose decreased from Tertile 1 (102.68 mg/dL) to Tertile 3 (97.75 mg/dL) (*P* < 0.001), and glycated hemoglobin followed a similar trend (*P* < 0.001).

Interestingly, an increase in LDL levels was found across tertiles, from 22.22% in Tertile 1 to 26.56% in Tertile 3 (*P* < 0.001). Similar trends were observed in TC and HDL levels, which increased from 26.82% in Tertile 1 to 29.55% in Tertile 3, and from 35.21% in Tertile 1 to 37.91% in Tertile 3, respectively (*P* < 0.001). A reversed association was observed in TG levels, decreasing from 36.06% in Tertile 1 to 34.33% in Tertile 3 (*P* < 0.001). Moreover, the prevalence of diabetes decreased significantly across tertiles, from 22.38% in Tertile 1 to 15.59% in Tertile 3 (*P* < 0.001).

These findings suggest that a higher PWR is associated with younger age, healthier behaviors, and better metabolic health, despite some increases in LDL and TC levels.

### 3.2 The correlation between PWR and diabetes

In the logistic regression models, elevated PWR levels were inversely associated with diabetes risk (**Table 2**). As a continuous variable, increased PWR values decreased the risk of diabetes, with the ORs ranging from 0.988–0.991 (all *P* < 0.001). Compared with the lowest PWR tertiles (T1), ORs of the T2 group and T3 group consistently decreased (*P* for trend < 0.001). For example, a 0.68-fold (95% CI: 0.60–0.78, *P* < 0.001) risk of diabetes was detected for the T3 group in the final model. Therefore, we found a robust inverse correlation between escalating PWR and decreasing diabetes risk among elderly Chinese individuals.

**Table 2:** The association of PWR with diabetes.

Models	PWR (continuous)	PWR (as tertiles)			
	OR (95% CI)	T1 (reference)	T2 group OR (95% CI)	T3 group OR (95% CI)	*P* for trend
Model 1	0.988 (0.985–0.992) ***	1.00	0.83 (0.74–0.93) ***	0.64 (0.57–0.72) ***	<0.001
Model 2	0.990 (0.986–0.993) ***	1.00	0.82 (0.73–0.92) ***	0.67 (0.59–0.75) ***	<0.001
Model 3	0.990 (0.986–0.994) ***	1.00	0.83 (0.74–0.93) ***	0.67 (0.59–0.76) ***	<0.001
Model 4	0.991 (0.987–0.995) ***	1.00	0.83 (0.73–0.93) **	0.68 (0.60–0.78) ***	<0.001

The T1 group was set as the reference group. **P < 0.01; ***P < 0.001. Model 1 – crude model; Model 2 - adjusting for age, gender, marital status, and BMI; Model 3 – further adjusting for cigarette and alcohol consumption, sleep duration and afternoon nap; Model 4 – adjusting for depression, hypertension and hyperuricemia, low-density lipoprotein, high-density lipoprotein, total cholesterol, and triglycerides. PWR: platelet to white blood cell ratio; T, tertile.

### 3.3 Subgroup analysis

The difference in effects between the distinct subgroup was tested using subgroup analysis (**Table 3**). The analysis revealed that participants in Tertile 3 exhibited lower odds of diabetes across various subgroups compared to those in Tertile 1. Specifically, individuals aged 60–70 years in Tertile 3 had significantly lower odds of diabetes (OR = 0.55, 95% CI: 0.44–0.68, *P* < 0.001). Similar findings were found in males (OR = 0.65, 95% CI: 0.53–0.80, *P* < 0.001) and females (OR = 0.70, 95% CI: 0.59–0.83, *P* < 0.001).

**Table 3:** Subgroup and interactive analysis.

Subgroups	T1	T2	*P* value	T3	*P* value	*P* for interaction
Age groups (years)						0.087
<50	1.00	0.81 (0.57–1.15)	0.233	0.76 (0.54–1.08)	0.127	
50–60	1.00	0.79 (0.63–0.98)	0.034	0.80 (0.64–1.01)	0.056	
60–70	1.00	0.82 (0.67–0.99)	0.039	0.55 (0.44–0.68)	<0.001	
>70	1.00	0.96 (0.73–1.26)	0.782	0.76 (0.56–1.03)	0.076	
Gender						0.774
Male	1.00	0.84 (0.71–0.99)	0.046	0.65 (0.53–0.80)	<0.001	
Female	1.00	0.82 (0.69–0.97)	0.022	0.70 (0.59–0.83)	<0.001	
Marital Status						0.524
Married/cohabitating	1.00	0.81 (0.71–0.92)	0.001	0.67 (0.59–0.78)	<0.001	
Others	1.00	0.93 (0.70–1.24)	0.645	0.75 (0.55–1.02)	0.066	
BMI (Kg/m^2^)						0.176
<18.5	1.00	0.98 (0.53–1.81)	0.951	0.65 (0.32–1.32)	0.237	
18.5–24.0	1.00	0.85 (0.69–1.03)	0.103	0.73 (0.60–0.90)	0.003	
24.0–28.0	1.00	0.92 (0.76–1.11)	0.384	0.73 (0.59–0.89)	0.003	
≥28.0	1.00	0.60 (0.45–0.79)	<0.001	0.54 (0.40–0.73)	<0.001	
Cigarette consumption						0.559
Current smoker	1.00	0.96 (0.76–1.20)	0.690	0.77 (0.58–1.01)	0.055	
Non-smoker	1.00	0.79 (0.67–0.93)	0.004	0.67 (0.57–0.79)	<0.001	
Ex-smoker	1.00	0.80 (0.59–1.08)	0.140	0.61 (0.42–0.87)	0.007	
Alcohol consumption						0.625
Drink more than once a month	1.00	0.79 (0.63–1.00)	0.053	0.59 (0.45–0.77)	<0.001	
Drink less than once a month	1.00	0.79 (0.51–1.24)	0.305	0.84 (0.52–1.35)	0.465	
None of These	1.00	0.85 (0.73–0.99)	0.032	0.71 (0.61–0.83)	<0.001	
Sleep duration (hours)						0.641
0–6	1.00	0.86 (0.73–1.01)	0.067	0.66 (0.55–0.79)	<0.001	
6–8	1.00	0.81 (0.66–0.98)	0.031	0.70 (0.57–0.85)	0.001	
>8	1.00	0.76 (0.51–1.13)	0.175	0.78 (0.52–1.17)	0.232	
Afternoon nap						0.382
No	1.00	0.82 (0.68–1.00)	0.051	0.76 (0.62–0.93)	0.008	
Yes	1.00	0.83 (0.71–0.97)	0.016	0.64 (0.54–0.75)	<0.001	
Depression						0.919
No	1.00	0.84 (0.73–0.97)	0.021	0.71 (0.60–0.83)	<0.001	
Yes	1.00	0.81 (0.66–1.00)	0.046	0.66 (0.53–0.81)	<0.001	
Hypertension						0.090
No	1.00	0.86 (0.72–1.03)	0.101	0.80 (0.67–0.96)	0.017	
Yes	1.00	0.80 (0.68–0.94)	0.006	0.59 (0.49–0.70)	<0.001	
Hyperuricemia						0.219
No	1.00	0.86 (0.76–0.98)	0.026	0.69 (0.60–0.80)	<0.001	
Yes	1.00	0.65 (0.47–0.89)	0.008	0.67 (0.47–0.96)	0.028	
LDL (mg/dL)						0.890
≤120	1.00	0.85 (0.74–0.97)	0.018	0.71 (0.61–0.82)	<0.001	
>120	1.00	0.77 (0.61–0.97)	0.025	0.62 (0.49–0.80)	<0.001	
Total cholesterol (mg/dL)						0.673
≤200	1.00	0.86 (0.74–0.99)	0.038	0.73 (0.62–0.85)	<0.001	
>200	1.00	0.78 (0.63–0.96)	0.018	0.62 (0.50–0.77)	<0.001	
Reduced HDL						0.014
No	1.00	0.96 (0.82–1.14)	0.657	0.75 (0.62–0.89)	0.002	
Yes	1.00	0.70 (0.59–0.83)	<0.001	0.62 (0.51–0.74)	<0.001	
Elevated triglycerides						0.098
No	1.00	0.88 (0.75–1.04)	0.148	0.79 (0.66–0.94)	0.007	
Yes	1.00	0.77 (0.65–0.92)	0.004	0.59 (0.49–0.71)	<0.001	

During the regression analysis, the subgroup variable was not adjusted in the full model. A multiplicative term was constructed to test the interactive effects. The T1 group was set as the reference group.

Participants in Tertile 3 also had significantly lower risks of diabetes among those with a BMI ≥ 28.0 kg/m² (OR = 0.54, 95% CI: 0.40–0.73, *P* < 0.001), non-smokers (OR = 0.67, 95% CI: 0.57–0.79, *P* < 0.001) and ex-smokers (OR = 0.61, 95% CI: 0.42–0.87, *P* = 0.007), and participants who drank alcohol more than once a month (OR = 0.59, 95% CI: 0.45–0.77, *P* < 0.001). Additionally, participants who slept 6–8 hours per night (OR = 0.70, 95% CI: 0.57–0.85, *P* = 0.001) or took afternoon naps (OR = 0.64, 95% CI: 0.54–0.75, *P* < 0.001) in Tertile 3 showed a decreased risk of diabetes. Furthermore, participants without depression (OR = 0.71, 95% CI: 0.60–0.83, *P* < 0.001), hypertension (OR = 0.59, 95% CI: 0.49–0.70, *P* < 0.001), or hyperuricemia (OR = 0.69, 95% CI: 0.60–0.80, *P* < 0.001) in Tertile 3 reported negative associations.

Moreover, participants with LDL levels > 120 mg/dL (OR=0.62, 95% CI: 0.49–0.80, *P* < 0.001), total cholesterol > 200 mg/dL (OR = 0.62, 95% CI: 0.50–0.77, *P* < 0.001), reduced HDL (OR = 0.62, 95% CI: 0.51–0.74, *P* < 0.001), and elevated triglycerides (OR = 0.59, 95% CI: 0.49–0.71, *P* < 0.001) in Tertile 3 had substantially lower risks of diabetes.

The interaction analysis revealed significant moderation effects for HDL levels (*P* for interaction = 0.014), indicating that the relationship between PWR tertiles and diabetes risk varies significantly between participants with normal and reduced HDL levels.

### 3.4 The nonlinear relationship between PWR and diabetes

**Figure 2:**
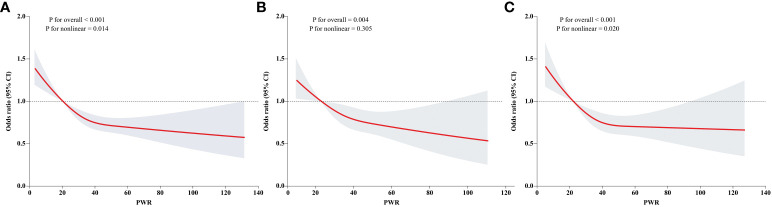
Dose response association of PWR with diabetes. Dose response association of PWR with diabetes was explored by the RCS regression. The linear and non-linear associations in the overall population, males, and females were displayed in **(A–C)**, respectively.

The smooth curve was fitted to present the nonlinear association between PWR and diabetes (**Figure 2**). We identified a decreasing trend in the total participants (**Figure 2A**), males only (**Figure 2B**), and females only (**Figure 2C**), respectively (*P* for overall < 0.01). Specifically, we found a nonlinear association in the total participants and females (*P* for nonlinear: 0.014 and 0.020, respectively). However, we cannot discern a nonlinear association in males (*P* for nonlinear: 0.305).

### 3.5 The correlation between PWR and diabetes in individuals with prediabetes

To elucidate the correlation between PWR and diabetes in participants with prediabetes, we further excluded individuals with normoglycemia. Individuals with prediabetes were set as reference. A negative association was observed regardless of whether PWR was treated as continuous or tertiles (**Table 4**). Additionally, each incremental unit of PWR corresponded to an OR of 0.987 in model 1, 0.988 in model 2, 0.989 in model 3, and 0.990 in model 4, respectively (all *P* < 0.001). In relation to the lowest PWR tertiles (T1), ORs of the T2 group and T3 group consistently decreased (*P* for trend < 0.001). After adjusting for all the covariates, 0.80-fold (95% CI = 0.71–0.91, *P* < 0.001) and 0.68-fold (95% CI = 0.59–0.77, *P* < 0.001) risks were detected for the T2 and T3 groups.

**Table 4:** The association of PWR with diabetes in individuals with prediabetes.

Models	PWR (continuous)	PWR (as tertiles)			
	OR (95% CI)	T1 (reference)	T2 group OR (95% CI)	T3 group OR (95% CI)	*P* for trend
Model 1	0.987 (0.984–0.991) ***	1.00	0.79 (0.70–0.89) ***	0.62 (0.55–0.70) ***	<0.001
Model 2	0.988 (0.985–0.992) ***	1.00	0.79 (0.70–0.89) ***	0.64 (0.57–0.73) ***	<0.001
Model 3	0.989 (0.985–0.993) ***	1.00	0.79 (0.70–0.90) ***	0.65 (0.57–0.74) ***	<0.001
Model 4	0.990 (0.986–0.994) ***	1.00	0.80 (0.71–0.91) ***	0.68 (0.59–0.77) ***	<0.001

To investigate the association of PWR with diabetes in individuals with prediabetes, individuals with normoglycemia were excluded. ***P < 0.001. Model 1 – crude model; Model 2 - adjusting for age, gender, marital status, and BMI; Model 3 – further adjusting for cigarette and alcohol consumption, sleep duration and afternoon nap; Model 4 – adjusting for depression, hypertension and hyperuricemia, low-density lipoprotein, high-density lipoprotein, total cholesterol, and triglycerides.

### 3.6 Sensitivity analysis

First, we examined the association between binary PWR defined by threshold values of dose-relationship links, and the risk of diabetes. We observed a 0.80-fold (95% CI: 0.68–0.94, *P* < 0.001) decrease in risk of diabetes for those with PWR ≥ 20.421 in the full model (**Table 5**). Second, we modeled the adjusted logistic regression using PWR as binary or quartiles (**Table 6**). Analogue findings were observed for both binary PWR and quartiles. For example, individuals with PWR above the median had a 0.78-fold (95% CI: 0.71–0.87, *P* < 0.001) decrease in risk of diabetes in relative to those with lower PWR values in the model 4. In quartile analysis, the T4 group exhibited a 0.67-fold (*P* < 0.05) reduction in diabetes risk relative to the T1 group, as per Model 4.

**Table 5:** The association between PWR and diabetes (as binary according to RCS regression).

Models	PWR < 20.421	PWR ≥ 20.421	*P*
	Reference	OR (95% CI)	
Model 1	1.00	0.80 (0.69–0.93)	0.004
Model 2	1.00	0.79 (0.68–0.93)	0.004
Model 3	1.00	0.81 (0.69–0.95)	0.008
Model 4	1.00	0.80 (0.68–0.94)	0.006

In the overall population, a non-linear association between PWR and diabetes was detected, with an inflection point of 20.421. Thus, we recoded the PWR as a binary variable according to the dose-response analysis. Model 1 – crude model; Model 2 - adjusting for age, gender, marital status, and BMI; Model 3 – further adjusting for cigarette and alcohol consumption, sleep duration and afternoon nap; Model 4 – adjusting for depression, hypertension and hyperuricemia, low-density lipoprotein, high-density lipoprotein, total cholesterol, and triglycerides.

**Table 6:** The association between PWR and diabetes (as binary or quartiles).

Models	B1/Q1 group Reference	Binary OR (95% CI)	Q2 group	Q3 group	Q4 group	*P* for trend
			OR (95% CI)	OR (95% CI)	OR (95% CI)	
Model 1	1.00	0.75 (0.68–0.83) ***	0.86 (0.75–0.98) *	0.78 (0.68–0.89) ***	0.62 (0.54–0.71) ***	<0.001
Model 2	1.00	0.77 (0.69–0.85) ***	0.84 (0.74–0.96) *	0.76 (0.66–0.87) ***	0.65 (0.56–0.75) ***	<0.001
Model 3	1.00	0.77 (0.70–0.86) ***	0.85 (0.75–0.97) *	0.77 (0.67–0.88) ***	0.65 (0.57–0.76) ***	<0.001
Model 4	1.00	0.78 (0.71–0.87) ***	0.86 (0.75–0.98) *	0.77 (0.67–0.89) ***	0.67 (0.58–0.78) ***	<0.001

As a sensitivity analysis, the participants were also grouped according to the median or quartiles of PWR. Individuals with PWR < median or in the Q1 group were set as reference. *P < 0.05; ***P < 0.001. Model 1 – crude model; Model 2 - adjusting for age, gender, marital status, and BMI; Model 3 – further adjusting for cigarette and alcohol consumption, sleep duration and afternoon nap; Model 4 – adjusting for depression, hypertension and hyperuricemia, low-density lipoprotein, high-density lipoprotein, total cholesterol, and triglycerides.

Same analytic methods were applied in the data without interpolation. First, we observed a persistent decrease in the association between PWR and diabetes, although no evidence of a nonlinear relationship (all *P* for overall < 0.05) (**Supplementary Figure S1**). We founded an evident association of PWR, whether treated as continuous or tertiles, with diabetes. For example, an increase of 1 unit in PWR was associated with a 0.991-fold (95% CI: 0.987–0.997, *P* < 0.001) decrease in risk of diabetes (**Supplementary Table S1**). Similarly, decreased risks of diabetes were detected in nearly all the subgroups (**Supplementary Table S2**). Lastly, increased PWR was also negatively associated with the risk of diabetes, with the ORs of 0.991 (95% CI: 0.987–0.995, *P* < 0.001) as continuous (**Supplementary Table S3**).

## 4 Discussion

As far as we know, this study represents the inaugural endeavor to examine the cross-sectional relationships between PWR and diabetes and the progression from prediabetes to diabetes based on a national survey in China. Our findings suggest that individuals with higher PWR exhibit a reduced risk of developing diabetes among the senior demographic in China.

In the baseline survey, individuals with elevated PWR were generally younger, predominantly female, with normal body weight, non-smokers, abstainers from alcohol, longer sleep duration, fewer naps, and demonstrated better lipid profiles, blood pressure, blood glucose, and uric acid. It has been found that type 2 diabetes patients commonly experience complications, which are more prevalent in males and older individuals (33). The most common complications include hypertension (82.1%), followed by overweight/obesity (78.2%) and hyperlipidemia (77.2%) (33). This suggests that individuals with higher PWR may have healthier lifestyles and lower risks of diabetes or prediabetes.

Consistent with our research findings, multiple studies have reported associations between PWR and prognosis in various malignancies and inflammatory conditions (25). Elevated PWR is significantly negatively correlated with overall survival rates among patients experiencing acute-on-chronic liver failure (34). Similarly, decreased PWR is autonomously linked with adverse outcomes among patients with pancreatic cancer (29) and HBV-associated decompensated cirrhosis (24). Moreover, patients with aneurysmal subarachnoid hemorrhage who have preoperative low PWR are at increased risk of developing postoperative pneumonia (28).

The mechanism explaining the relationship between PWR and diabetes still needs to be elucidated. Insufficient insulin secretion and insulin resistance are two crucial factors in the pathogenesis of Type 2 diabetes (35). WBC count serves as a marker of inflammation, mediating the body’s immune response (36). Insulin resistance is associated with peripheral WBC count, indicating that elevated WBC count is a predictor of insulin resistance (37). The precise mechanism behind the association between WBC count and insulin resistance remains unclear. Several studies have indicated that interleukin-6, primarily produced in adipose tissue, acts as a significant factor in WBC differentiation and is linked to insulin resistance (38). Additionally, hormones serve as a potential connection between WBCs and insulin sensitivity. Many hormones have receptors on WBC surfaces and influence their development and maturity. Among these hormones, insulin, cortisol, and sex hormones are associated with insulin resistance (37).

Platelet dysfunction is pivotal in the occurrence and progression of vascular complications in diabetes. Platelet activation may represent an early occurrence in the natural progression of diabetes (39). Research has shown that mean platelet volume is notably elevated in both diabetic and impaired fasting glucose groups compared to controls. Furthermore, there was a positive correlation between mean platelet volume and platelet mass concerning fasting glucose and HbA1c levels in both diabetic and impaired fasting glucose groups (40, 41). Increased platelet aggregation has been observed in diabetes since as early as 1965 (42), and subsequent studies have consistently shown increased platelet degranulation and production of thromboxane derivatives, leading to additional activation of platelets (43, 44). Additionally, platelet-mediated vasodilation is impaired in diabetes (45), and platelets from diabetic patients exhibit reduced responsiveness to endogenous anti-aggregating agents like prostaglandin I2 and nitric oxide (46, 47). Notably, numerous studies have already suggested a link between poor glycemic control and increased platelet activity (48–50). The altered platelet function observed in individuals with diabetes may involve various mechanisms, with metabolic changes, oxidative stress, and endothelial dysfunction playing significant roles (51). However, our study revealed a positive correlation between decreased PWR and increased risk of diabetes. This association may be attributed to the propensity of platelets to adhere to vessel walls at high blood glucose concentrations in diabetic individuals, resulting in a decrease in peripheral blood platelets (39). Additionally, poor glycemic control may lead to liver damage (52), as the liver is a crucial organ for producing thrombopoietin, a platelet-stimulating factor, which could further contribute to decreased platelet count (53). Indeed, for individuals with normal blood glucose levels, an elevated PWR might correlate with improved platelet function, which is vital for preserving vascular health, hemostatic function, and immune system functionality (25).

The effect of platelet and WBC counts can be simultaneously assessed by the PWR (54). The interaction between platelets and WBCs has been implicated in the pathogenesis of numerous diseases (25). Inflammatory pathways are considered potential mediators in the pathogenesis of diabetes (55). Platelets affect other blood cells by releasing chemokines and membrane ligands and facilitating leukocyte-platelet aggregates in the peripheral blood (56). Consequently, PWR can reflect the degree of inflammation, and a significant association of PWR with diabetes may indicate a more prominent effect of PLTs than WBCs (54). During inflammation, the proportion of larger platelets tends to rise, likely due to the production of factors that encourage coagulation and inflammation, as well as the release of platelets stored in the spleen (57). Concurrently, these platelets are swiftly recruited to the site of inflammation, where they may become activated and depleted, potentially explaining the reduced mean platelet volume observed in patients experiencing inflammation (58). Additionally, the spleen is a major immune organ that stores and filters blood cells (59). Diabetes patients often have a chronic inflammatory state, which may lead to an increase in WBCs (60). If spleen function is abnormal or impaired, it could affect the storage and release of WBCs, thus influencing PWR (61). Furthermore, the bone marrow is the primary site for the production of platelets and WBCs (62). The chronic inflammatory state induced by diabetes can stimulate the bone marrow to produce more WBCs (63). Overactive bone marrow could lead to an elevated WBC count, thereby decreasing PWR. In diabetes, especially with poor glycemic control, bone marrow might increase platelet production (64). This increase could temporarily raise PWR, but long-term high blood glucose levels might lead to bone marrow exhaustion, reducing platelet production and thus affecting PWR (65). Therefore, we propose that a low PWR may reflect the severity of inflammation and potentially impact the risk of diabetes. Further research is needed to elucidate the underlying mechanisms of this association. This research utilized information from the CHARLS database, which offers the advantage of a substantial sample size across multiple regions. However, there are several limitations to consider. Firstly, as the data is sourced from a public database, there is a lack of control over the original data quality and detailed background information on the study participants, necessitating further validation of the research findings through clinical practice. Secondly, the diagnostic criteria for diabetes were not comprehensive, and there was a deficiency in related clinical manifestation data. Grouping was solely based on fasting blood glucose and HbA1c levels, potentially leading to some false positive and false negative results. Furthermore, the study did not distinguish between type 1 and type 2 diabetes, warranting further investigation into the correlation between different types of diabetes and PWR. Finally, the cross-sectional association should be further verified in future longitudinal surveys.

## 5 Conclusions

This cross-sectional survey discloses that elevated PWR is significantly associated with decreased risks of diabetes. There are non-linear associations of PWR and diabetes in the overall population and females, but not in males. The dose-response association between PWR and diabetes indicates that PWR holds potentials in early identification and prevention of diabetes. The role and mechanism of hematological indicators in predicting diabetes should be further investigated in future studies.

## Data availability statement

Publicly available datasets were analyzed in this study. This data can be found here: https://charls.pku.edu.cn/

## Ethics statement

The studies involving humans were approved by Peking University’s ethical review board (IRB number:00001052-11014). The studies were conducted in accordance with the local legislation and institutional requirements. The participants provided their written informed consent to participate in this study.

## Author contributions

FL: Conceptualization, Formal analysis, Investigation, Methodology, Project administration, Software, Validation, Writing – original draft. TW: Conceptualization, Data curation, Formal analysis, Investigation, Methodology, Writing – review & editing. SW: Conceptualization, Methodology, Project administration, Validation, Writing – review & editing. ZX: Data curation, Methodology, Software, Visualization, Writing – review & editing. YH: Conceptualization, Project administration, Resources, Supervision, Writing – review & editing.
