# Supplementary material for: The association of platelet to white blood cell ratio with diabetes: a nationwide survey in China
Source: Front Endocrinol (Lausanne). 2024 Jun 18;15:1418583. doi: 10.3389/fendo.2024.1418583 (PMC11217324; doi:10.3389/fendo.2024.1418583)
Supplement: Supplementary file 1 [file DataSheet_1.docx]

| **Table S1 The association of PWR with diabetes in the raw dataset** | | | | | |
| --- | --- | --- | --- | --- | --- |
| **Models** | **PWR (continous)** | **PWR (as tertiles)** | | | |
|  | **OR (95% CI)** | **T1**  **(reference)** | **T2 group**  **OR (95% CI)** | **T3 group**  **OR (95% CI)** | ***P* for trend** |
| Model 1 | 0.988 (0.985-0.992) *** | 1.00 | 0.83 (0.74-0.93) *** | 0.64 (0.57-0.72) *** | <0.001 |
| Model 2 | 0.990 (0.986-0.994) *** | 1.00 | 0.83 (0.73-0.93) *** | 0.67 (0.59-0.76) *** | <0.001 |
| Model 3 | 0.990 (0.986-0.994) *** | 1.00 | 0.83 (0.74-0.94) ** | 0.66 (0.58-0.76) *** | <0.001 |
| Model 4 | 0.991 (0.987-0.996) *** | 1.00 | 0.83 (0.73-0.94) ** | 0.69 (0.60-0.79) *** | <0.001 |

The dataset was not interpolated by the random forest. The covariates had 0%-8.98% missing values. ** *P* < 0.01; *** *P* < 0.001. Model 1 – crude model; Model 2 – adjusting for age, gender, marital status, and BMI; Model 3 – further adjusting for cigarette and alcohol consumption, sleep duration and afternoon nap; Model 4 – adjusting for depression, hypertension and hyperuricemia, low-density lipoprotein, high-density lipoprotein, total cholesterol, and triglycerides.

| **Table S2: Subgroup and interactive analysis in the raw dataset** | | | | | | |
| --- | --- | --- | --- | --- | --- | --- |
| **Subgroups** | **T1** | **T2** | ***P* value** | **T3** | ***P* value** | ***P* for interaction** |
| **Age groups (years)** |  |  |  |  |  | 0.285 |
| <50 | 1.00 | 0.77 (0.54-1.10) | 0.154 | 0.73 (0.51-1.04) | 0.083 |  |
| 50-60 | 1.00 | 0.79 (0.62-0.99) | 0.049 | 0.79 (0.62-0.99) | 0.049 |  |
| 60-70 | 1.00 | 0.88 (0.71-1.08) | 0.021 | 0.58 (0.46-0.74) | <0.001 |  |
| >70 | 1.00 | 0.89 (0.65-1.22) | 0.465 | 0.76 (0.54-1.08) | 0.127 |  |
| **Gender** |  |  |  |  |  | 0.495 |
| Male | 1.00 | 0.82 (0.68-0.98) | 0.030 | 0.62 (0.50-0.77) | <0.001 |  |
| Female | 1.00 | 0.86 (0.71-1.03) | 0.099 | 0.74 (0.62-0.89) | 0.001 |  |
| **Marital Status** |  |  |  |  |  | 0.783 |
| Married/cohabitating | 1.00 | 0.82 (0.71-0.94) | 0.006 | 0.69 (0.59-0.80) | <0.001 |  |
| Others | 1.00 | 0.89 (0.65-1.23) | 0.492 | 0.75 (0.53-1.07) | 0.109 |  |
| **BMI (Kg/m^2^)** |  |  |  |  |  | 0.123 |
| <18.5 | 1.00 | 0.91 (0.45-1.83) | 0.185 | 0.54 (0.22-1.30) | 0.029 |  |
| 18.5-24.0 | 1.00 | 0.86 (0.69-1.07) | 0.464 | 0.78 (0.63-0.98) | 0.001 |  |
| 24.0-28.0 | 1.00 | 0.93 (0.76-1.13) | <0.001 | 0.70 (0.56-0.87) | 0.001 |  |
| ≥28.0 | 1.00 | 0.58 (0.43-0.79) | 0.281 | 0.58 (0.42-0.79) | 0.061 |  |
| **Cigarette consumption** |  |  |  |  |  | 0.550 |
| Current smoker | 1.00 | 0.89 (0.70-1.13) | 0.345 | 0.75 (0.57-1.01) | 0.056 |  |
| Non-smoker | 1.00 | 0.82 (0.69-0.97) | 0.024 | 0.71 (0.60-0.84) | <0.001 |  |
| Ex-smoker | 1.00 | 0.82 (0.60-1.13) | 0.235 | 0.54 (0.36-0.80) | 0.002 |  |
| **Alcohol consumption** |  |  |  |  |  | 0.310 |
| Drink more than once a month | 1.00 | 0.76 (0.59-0.97) | 0.029 | 0.57 (0.43-0.76) | <0.001 |  |
| Drink less than once a month | 1.00 | 0.87 (0.54-1.41) | 0.576 | 1.05 (0.63-1.74) | 0.859 |  |
| None of These | 1.00 | 0.86 (0.73-1.01) | 0.060 | 0.71 (0.60-0.84) | <0.001 |  |
| **Sleep duration (hours)** |  |  |  |  |  | 0.419 |
| 0-6 | 1.00 | 0.88 (0.73-1.05) | 0.145 | 0.66 (0.54-0.80) | <0.001 |  |
| 6-8 | 1.00 | 0.81 (0.66-1.00) | 0.053 | 0.72 (0.58-0.90) | 0.004 |  |
| >8 | 1.00 | 0.66 (0.43-1.02) | 0.061 | 0.75 (0.48-1.17) | 0.202 |  |
| **Afternoon nap** |  |  |  |  |  | 0.223 |
| No | 1.00 | 0.83 (0.67-1.03) | 0.087 | 0.81 (0.65-1.01) | 0.057 |  |
| Yes | 1.00 | 0.83 (0.70-0.98) | 0.024 | 0.63 (0.52-0.75) | <0.001 |  |
| **Depression** |  |  |  |  |  | 0.899 |
| No | 1.00 | 0.83 (0.71-0.97) | 0.021 | 0.71 (0.60-0.84) | <0.001 |  |
| Yes | 1.00 | 0.84 (0.67-1.05) | 0.123 | 0.66 (0.53-0.83) | <0.001 |  |
| **Hypertension** |  |  |  |  |  | 0.240 |
| No | 1.00 | 0.82 (0.68-0.99) | 0.043 | 0.77 (0.64-0.94) | 0.009 |  |
| Yes | 1.00 | 0.83 (0.70-0.99) | 0.045 | 0.62 (0.51-0.75) | <0.001 |  |
| **Hyperuricemia** |  |  |  |  |  | 0.654 |
| No | 1.00 | 0.85 (0.74-0.98) | 0.025 | 0.70 (0.61-0.81) | <0.001 |  |
| Yes | 1.00 | 0.72 (0.51-1.01) | 0.059 | 0.67 (0.45-0.99) | 0.048 |  |
| **LDL (mg/dL)** |  |  |  |  |  | 0.878 |
| ≤120 | 1.00 | 0.83 (0.71-0.96) | 0.015 | 0.71 (0.61-0.84) | <0.001 |  |
| >120 | 1.00 | 0.81 (0.63-1.05) | 0.111 | 0.63 (0.49-0.82) | 0.001 |  |
| **Total cholesterol (mg/dL)** |  |  |  |  |  | 0.869 |
| ≤200 | 1.00 | 0.85 (0.72-0.99) | 0.040 | 0.72 (0.61-0.86) | <0.001 |  |
| >200 | 1.00 | 0.80 (0.64-0.99) | 0.049 | 0.65 (0.51-0.81) | <0.001 |  |
| **Reduced HDL** |  |  |  |  |  | 0.091 |
| No | 1.00 | 0.92 (0.77-1.10) | 0.384 | 0.75 (0.62-0.91) | 0.004 |  |
| Yes | 1.00 | 0.73 (0.61-0.89) | 0.001 | 0.62 (0.51-0.76) | <0.001 |  |
| **Elevated triglycerides** |  |  |  |  |  | 0.280 |
| No | 1.00 | 0.85 (0.71-1.01) | 0.065 | 0.77 (0.64-0.93) | 0.006 |  |
| Yes | 1.00 | 0.82 (0.68-0.99) | 0.041 | 0.62 (0.51-0.76) | <0.001 |  |

The dataset was not interpolated by the random forest. The covariates had 0%-8.98% missing values. The T1 group was set as the reference group. ** *P* < 0.01; *** *P* < 0.001. Model 1 – crude model; Model 2 - adjusting for age, gender, marital status, and BMI; Model 3 – further adjusting for cigarette and alcohol consumption, sleep duration and afternoon nap; Model 4 – adjusting for depression, hypertension and hyperuricemia, low-density lipoprotein, high-density lipoprotein, total cholesterol, and triglycerides. PWR: platelet to white blood cell ratio; T: tertile.

| **Table S3 The association of PWR with diabetes in individuals with prediabetes in the raw dataset** | | | | | |
| --- | --- | --- | --- | --- | --- |
| **Models** | **PWR (continous)** | **PWR (as tertiles)** | | | |
|  | **OR (95% CI)** | **T1**  **(reference)** | **T2 group**  **OR (95% CI)** | **T3 group**  **OR (95% CI)** | ***P* for trend** |
| Model 1 | 0.987 (0.984-0.991) *** | 1.00 | 0.79 (0.70-0.89) *** | 0.62 (0.55-0.70) *** | <0.001 |
| Model 2 | 0.989 (0.985-0.992) *** | 1.00 | 0.79 (0.70-0.90) *** | 0.64 (0.56-0.73) *** | <0.001 |
| Model 3 | 0.989 (0.985-0.993) *** | 1.00 | 0.80 (0.70-0.91) *** | 0.64 (0.56-0.73) *** | <0.001 |
| Model 4 | 0.991 (0.987-0.995) *** | 1.00 | 0.81 (0.70-0.92) ** | 0.68 (0.59-0.79) *** | <0.001 |

The dataset was not interpolated by the random forest. The covariates had 0%-8.98% missing values. ** *P* < 0.01, *** *P* < 0.001. Model 1 – crude model; Model 2 - adjusting for age, gender, marital status, and BMI; Model 3 – further adjusting for cigarette and alcohol consumption, sleep duration and afternoon nap; Model 4 – adjusting for depression, hypertension and hyperuricemia, low-density lipoprotein, high-density lipoprotein, total cholesterol, and triglycerides.

**

**

**Figure S1: Dose response association of PWR with diabetes in the raw dataset**

The dataset was not interpolated by the random forest. The covariates had 0%-8.98% missing values. Dose response association of PWR with diabetes was explored by the RCS regression. The linear and non-linear associations in the overall population, males, and females were displayed in Figure S1A, S1B, and S1C, respectively.
